# Custo-Efetividade Relacionado aos Dispositivos Cardíacos Eletrônicos Implantáveis: Uma Preocupação Sempre Constante

**DOI:** 10.36660/abc.20240185

**Published:** 2024-04-17

**Authors:** Luiz Eduardo Montenegro Camanho

**Affiliations:** 1 Hospital Pró-Cardíaco Rio de Janeiro RJ Brasil Hospital Pró-Cardíaco, Rio de Janeiro, RJ – Brasil

**Keywords:** Marca-Passo Artificial, Análise de Custo-Efetividade, pneumotórax, Hematoma

Os dispositivos cardíacos eletrônicos implantáveis (DCEIs) desempenham um papel crucial nos portadores de bradiarritmias, com comprovada eficácia na melhora da qualidade de vida e na manutenção da própria vida, sendo inquestionável a sua utilização na prática clínica. No entanto, a tecnologia empregada e suas possíveis complicações representam um custo considerável junto as agências reguladoras e as respectivas fontes pagadoras. Se por um lado, não se questiona o benefício dos DCEIs em diferentes cenários clínicos, por outro, o custo associado, incluindo as possíveis complicações, também devem ser motivo de reflexão.

O custo inicial relacionado ao procedimento é elevado assim como o custo relacionado a possíveis complicações, tais como, pneumotórax, hemotórax, desposicionamento de eletrodo, infecção e complicações clínicas em geral.^1-4^

Esta questão já vem sendo observada e investigada de longa data. Na década de 90, Ferguson et al. demonstraram que os custos relacionados as complicações observadas em 1.031 implantes de marca-passo e 105 cardioversores-desfibriladores implantáveis excederam o valor reembolsado pela fonte pagadora, já salientando a dimensão e o desafio deste problema.^5^

Ludwig et al. apresentaram os resultados da ocorrência de complicações e custos relacionados aos procedimentos de implante de marca-passo ou troca de gerador em uma coorte de 12.922 pacientes, seguidos por 12 meses após a intervenção. A taxa de complicação foi de 12% e, principalmente relacionada à loja do marca-passo e aos eletrodos. Em 36 meses pós complicação, o custo médio foi €4627 acima do que nos pacientes sem complicação. Os autores concluíram que a ocorrência de complicações pós-procedimento representa um substancial custo para os pacientes e sistema de saúde.^6^

Clémenty et al.^7^ desenvolveram um estudo econômico nacional francês sobre complicações e custos relacionados ao implante de marca-passo no ano de 2012. O racional da pesquisa é que os marca-passos *leadless* (sem eletrodo) minimizam a taxa de complicações clássicas relacionadas ao implante. De um total de 65.553 pacientes, 11.770 (18%) seriam elegíveis para implante de um marca-passo *leadless*. A taxa de complicação observada foi de 5,3% durante um período de acompanhamento de 3 anos e, em 89% dos casos, esteve relacionado ao eletrodo ou a loja do marca-passo. No total, o custo médio por paciente com complicação foi de €6,674 ± 3,867. Especificamente, o custo foi de €7,143 ± 2,685 para hematoma de loja, €5,123 *+* 2,676 para pneumotórax e €6,020 + 3,272 para complicações mecânicas. Os autores concluem que as complicações maiores relacionadas ao eletrodo e a loja ainda são comuns e, apresentam um importante impacto econômico com aumento expressivo dos custos para os sistemas de saúde.^7^ Neste aspecto, vale ressaltar que os custos iniciais do marca-passo *leadless* em nosso meio ainda são muito elevados e, que estudos posteriores, devem avaliar a relação custo-benefício desta estratégia como redutora real de custo a médio e longo prazo.

As complicações relacionadas ao implante de dispositivos eletrônicos aumentaram exponencialmente nos últimos anos, em função do envelhecimento da população e da expansão das indicações. Entre 1993 e 2009, a taxa de implante dos Estados Unidos aumentou de 46,7 para 61,6/ 100.000 indivíduos.^8^

O hematoma de loja é uma complicação frequente após o implante de DCEIs. Sridhar et al.^9^ descreveram a ocorrência desta complicação em 1.677 (2,1%) de um total de 78.751 pacientes submetidos a procedimentos de marca-passo durante o ano de 2010. Os autores demonstraram que o surgimento de hematoma pós-operatório aumentou o tempo de hospitalização (8,7 vs. 4,8 dias, p < 0.001), os custos da internação (48,815 vs. 34,324, p < 0.001) com aumento da mortalidade intra-hospitalar (2,0 vs. 0,7%, p < 0.001), quando comparado com pacientes que não desenvolveram este tipo de complicação. Como conclusão, é proposto que o hematoma de loja é uma complicação relativamente comum após implante de marca-passo e associada a desfechos clínicos e econômicos desfavoráveis.^9^

Nesta revista, Alves et al.^10^ descreveram uma interessante série de 1.223 pacientes consecutivos submetidos a implante inicial (n= 634) ou troca do gerador de pulsos (n= 589). O objetivo foi determinar as taxas de readmissões hospitalares e complicações após implante de marca-passo ou troca de gerador de pulsos e avaliar o impacto desses eventos nos custos anuais do tratamento sob a perspectiva do Sistema Único de Saúde. Os autores demonstraram que a presença de doença renal crônica, histórico de acidente vascular encefálico, tempo de permanência hospitalar, necessidade de cuidados intensivos pós-operatórios, complicações e readmissões hospitalares mostraram um impacto significativo sobre o custo anual total do tratamento e concluíram que idade, comorbidades, complicações pós-operatórias e readmissões hospitalares representaram fatores associados ao incremento do custo total anual do tratamento de pacientes com marca-passo.^10^

O trabalho em questão traz informações relevantes referentes aos custos relacionados aos DCEIs e, indiscutivelmente nos auxiliam na prática médica diária. Medidas profiláticas de redução de complicações devem ser incorporadas às práticas clínicas para trazer substancial ganho ao paciente a as fontes pagadoras. Esta ponderação deve sempre permear a boa prática médica.
