# Supplementary material for: Custo-Efetividade Relacionado aos Dispositivos Cardíacos Eletrônicos Implantáveis: Uma Preocupação Sempre Constante
Source: Arq Bras Cardiol. 2024 Apr 17;121(4):e20240185. [Article in Portuguese] doi: 10.36660/abc.20240185 (PMC11098566; doi:10.36660/abc.20240185)
Supplement: Translation

# Translation: Cost-Effectiveness Related to Implantable Electronic Cardiac Devices: An Ever-Constant Concern

**DOI:** 10.36660/abc.20240185i

**Authors:** Luiz Eduardo Montenegro Camanho

**Affiliations:** 1 Brazil Hospital Pró-Cardíaco, Rio de Janeiro, RJ – Brazil

**Keywords:** Artificial Pacemaker, Cost-Effectiveness Analysis, Pneumothorax, Hematoma

Implantable electronic cardiac devices (IECDs) play a crucial role in patients with bradyarrhythmias, with proven effectiveness in improving quality of life and maintaining life itself, and their use in clinical practice is unquestionable. However, the technology used and its possible complications represent a considerable cost to regulatory agencies and the respective paying sources. If, on the one hand, the benefit of IECDs in different clinical scenarios is not questioned, on the other hand, the associated cost, including possible complications, should also be a reason for reflection.

The initial cost related to the procedure is high, as is the cost related to possible complications, such as pneumothorax, hemothorax, electrode disposition, infection, and clinical complications in general.^1-4^

This issue has been observed and investigated for a long time. In the 90s, Ferguson et al. demonstrated that the costs related to complications observed in 1,031 pacemaker implants and 105 implantable cardioverter-defibrillators exceeded the amount reimbursed by the paying source, highlighting the dimension and challenge of this problem.^5^

Ludwig et al. presented the results of the occurrence of complications and costs related to pacemaker implantation or generator replacement procedures in a cohort of 12,922 patients, followed for 12 months after the intervention. The complication rate was 12% and mainly related to the pacemaker site and electrodes. At 36 months post-complication, the average cost was €4627 higher than in patients without complications. The authors concluded that the occurrence of post-procedure complications represents a substantial cost for patients and the healthcare system.^6^

Clémenty et al.^7^ developed a French national economic study on complications and costs related to pacemaker implantation in 2012. The rationale for the research is that leadless pacemakers minimize the rate of classic complications related to the implant. Of a total of 65,553 patients, 11,770 (18%) would be eligible for implantation of a leadless pacemaker. The complication rate observed was 5.3% during a 3-year follow-up period and, in 89% of cases, it was related to the lead or the pacemaker pocket. In total, the average cost per patient with a complication was €6,674 ± 3,867. Specifically, the cost was €7,143 ± 2,685 for pocket hematoma, €5,123 + 2,676 for pneumothorax and €6,020 + 3,272 for mechanical complications. The authors conclude that major complications related to the electrode and the pocket are still common and have an important economic impact with a significant increase in costs for health systems.^7^ In this aspect, it is worth highlighting that the initial costs of the leadless pacemaker in our country are still very high, and further studies should evaluate the cost-benefit ratio of this strategy as a real cost reducer in the medium and long term.

Complications related to the implantation of electronic devices have increased exponentially in recent years, due to the aging of the population and the expansion of indications. Between 1993 and 2009, the United States implant rate increased from 46.7 to 61.6/100,000 individuals.^8^

Pocket hematoma is a common complication after IECD implantation. Sridhar et al.^9^ described the occurrence of this complication in 1,677 (2.1%) of a total of 78,751 patients undergoing pacemaker procedures in 2010. The authors demonstrated that the appearance of postoperative hematoma increased the length of hospitalization (8.7 vs. 4.8 days, p < 0.001), hospitalization costs (48.815 vs. 34.324, p < 0.001) with increased in-hospital mortality (2.0 vs. 0.7%, p < 0.001) when compared to patients who did not develop this type of complication. In conclusion, it is proposed that pocket hematoma is a relatively common complication after pacemaker implantation and is associated with unfavorable clinical and economic outcomes.^9^

In this journal, Alves et al.^10^ described an interesting series of 1,223 consecutive patients undergoing initial implantation (n= 634) or pulse generator exchange (n= 589). The objective was to determine the rates of hospital readmissions and complications after pacemaker implantation or pulse generator replacement and evaluate the impact of these events on annual treatment costs from the perspective of the Unified Health System. The authors demonstrated that the presence of chronic kidney disease, history of stroke, length of hospital stay, need for postoperative intensive care, complications, and hospital readmissions had a significant impact on the total annual cost of treatment and concluded that age, Comorbidities, postoperative complications, and hospital readmissions represented factors associated with the increase in the total annual cost of treating patients with pacemakers.^10^

The work in question provides relevant information regarding the costs related to IECDs and undoubtedly helps us in daily medical practice. Prophylactic measures to reduce complications must be incorporated into clinical practices to bring substantial benefits to the patient and paying sources. This consideration must always permeate good medical practice.
